# Vergence and Standing Balance in Subjects with Idiopathic Bilateral Loss of Vestibular Function

**DOI:** 10.1371/journal.pone.0066652

**Published:** 2013-06-18

**Authors:** Zoï Kapoula, Chrystal Gaertner, Qing Yang, Pierre Denise, Michel Toupet

**Affiliations:** 1 Group IRIS CNRS (Centre National de Recherche Scientifique), Centre d′Etudes de la SensoriMotricité UMR 8194, Université Paris V, Hôpital Européen Georges Pompidou, service d′Ophthalmologie, Paris, France; 2 INSERM, U7075, Caen, France; 3 Centre d′Explorations Otoneurologiques, Paris, France; Barrow Neurological Institute, United States of America

## Abstract

There is a natural symbiosis between vergence and vestibular responses. Deficits in vergence can lead to vertigo, disequilibrium, and postural instability. This study examines both vergence eye movements in patients with idiopathic bilateral vestibular loss, and their standing balance in relation to vergence. Eleven patients participated in the study and 16 controls. Bilateral loss of vestibular function was objectified with many tests; only patients without significant response to caloric tests, to video head impulse tests and without vestibular evoked myogenic potentials were included in the study.

**Vergence testing (from 8 patients and 15 controls):**

A LED display with targets at 20, 40, and 100 cm along the median plane was used to elicit vergence eye movements, recorded with the IRIS device.

**Standing balance (11 patients and 16 controls):**

Four conditions were run, each lasting 1 min: fixation of a LED at 40 cm (convergence of 9°), at 150 cm (convergence of 2.3°); this last condition was repeated with eyes closed. Comparison of the eyes closed-eyes open conditions at 150 cm allowed evaluation of the Romberg Quotient. In the forth condition, two LEDS, at 20 and at 100 cm, were light on, one after the other for 1 sec, causing the eyes to converge then diverge. Standing balance was recorded with an accelerometer placed at the back near the center of mass (McRoberts, Dynaport).

**Results:**

**Vergence:**

Relative to controls, convergence eye movements in patients showed significantly lower accuracy, lower mean velocity, and saccade intrusions of significantly higher amplitude.

**Balance:**

The normalized 90% area of body sway was significantly higher for patients than for controls for all conditions. Yet, similarly to controls, postural stability was better while fixating at near (sustained convergence) than at far, or while making active vergence movements. We argue that vestibular loss deteriorates convergence, but even deficient, convergence can be helpful for postural control.

## Introduction

Vergence eye movements allow adjusting of the angle of visual axis according to depth at which the object of interest is located. The eyes are moving in opposite directions to increase the angle for a near object (convergence) or to decrease it for a far object (divergence). Multiple stimuli participate in the triggering and execution of such movements: binocular disparity, blur and resulting accommodation, high level proximal cues, monocular depth cues such as visual perspective [1], [2]. Control of these complex eye movements involves the occipital-parietal-frontal cortex [3], [4], [5], [6], the superior colliculus [7], [8], the brainstem [9] and the cerebellum [10], [11], [12]. The cerebellum is involved in vergence control, both online (direct effect) [13], [14], [15] and offline (adaptation effect) [16], [17]. A recent brain imaging study provides further evidence for specific neural circuits supplying vergence versus saccades, namely within the frontal eye fields and the midbrain [18]. At the brainstem, convergence and divergence are supplied by specific phasic, tonic and phasic-tonic cells located in the mesencephalic reticular formation [6]. Thus divergence is not simply passive relaxation of convergence but a separate neurophysiological system.

Vergence eye movements are essential for single binocular fused vision, for perception of depth and for stereovision. The gain of vestibular response particularly of the translational vestibulo-ocular reflex (tVOR) is known to depend on viewing distance [19]; the gain increases for near vision and such increase could be mediated by vergence signals. Migliaccio et al. (2004) [20] has shown that vergence mediated modulation of the human horizontal vestibulo-ocular reflex (VOR) is eliminated by a partial peripheral gentamicin lesion. The authors propose that irregular afferents from the hair cells in the central zone of the crista provide the necessary signal used centrally to increase the gain of the VOR with vergence. Yet, at the central level, as the VOR is very rapidly triggered, it has been suggested that vergence involvement could act via the cerebellum, which learns to provide a predictive vergence input to VOR (see model proposed by Coenen) [21]. In line with such conceptual models about vergence-vestibular interaction, clinical studies showed that patients with progressive supranuclear palsy (PSP) show both vergence disorders and no increase of tVOR with near viewing [22], [23], [24]. Ramat and Zee [25] studied dynamics of oculomotor responses to abrupt head translation in healthy humans and reported scaling of tVOR with vergence angle; they also point that saccades are integral part of the tVOR scaled with viewing distance as well.

Over the recent years, our team studied extensively children and teenagers with vestibular type of syndromes (vertigo, dizziness and headaches) for whom no clinically assessed vestibular pathology could be found, yet abnormalities of eye movements particularly of vergence eye movements were present [26], [27], [28], [29], [30]. Keeping up with the concept of reciprocal interaction-symbiosis between vergence and vestibular function, the present study examines properties of convergence versus divergence eye movements in patients with idiopathic bilateral vestibular loss. We expected vergence abnormalities in such patients because of the loss of interactivity with the vestibular input. Evidence for boosting of vergence by vestibular function exists. Erkelens *et al.* [31] showed that vergence eye movements are faster and match better the target when the person moves the chest (body) to approach or get away from the target than when the eyes only are moving with head and body stabilized.

Another goal of the present study is to test postural control in relation to vergence in such patients. Paulus *et al* [32] have demonstrated that postural control improves while fixating at near than at far distance; such *stabilizing effect of proximity* would be mediated by increased angular size of the retinal drift at near distances. Yet, Kapoula and Lê [33] showed that the proximity stabilizing effect is also due to oculomotor signals related to increased convergence of the eyes at near. Indeed, convergent prisms used in order to make the eyes converge while the subjects were fixating at far distances resulted in an improvement in postural stability.

Therefore the questions asked in the present study were whether patients with idiopathic bilateral vestibular loss show the stabilization effect of proximity, hypothetically mediated by convergence, and whether active vergence movements, healthy or not, could still be useful for postural control.

## Materials and Methods

### Patients

**Figure 1:**
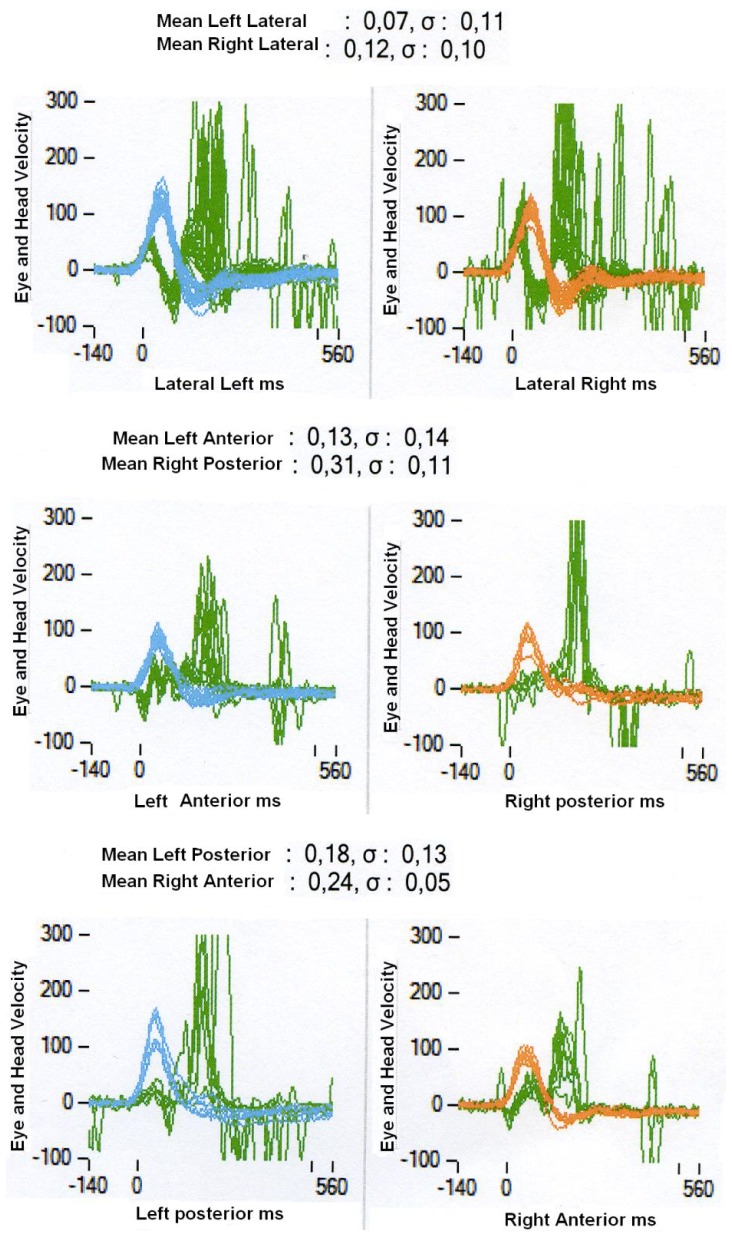
Example of patients Vestibular Head Impulse Test (VHIT). Traces in each panel show the head velocity (blue and orange) and eye velocity (green); data for stimulation of lateral, anterior and posterior left canals are shown on the left and the data for lateral, posterior and anterior right canals are shown on the right. The eye traces indicate very low gain relative to the head signal and multiple catch up saccades. The average mean gain and standard deviation are shown in each panel.

Eleven patients from 26 to 77 years (mean, **52±14.5**) with idiopathic bilateral loss of vestibular function were recruited in this study; all but two patients were below 60 years of age. These subjects were included as their results were not different from those of the remaining group. Patients suffered from bilateral idiopathic loss of vestibular function (BILVF) but not from hearing loss or associated neurological symptoms. All patients had acquired their vestibular loss at least 5 years prior to this experiment (with either simultaneous or sequential onset of BILVF), and none complained of persistent oscillopsia. Bilateral impairments of vestibular function were assessed with a battery of functional tests before the inclusion of the patients in the experiment. Video-nystagmography with bithermal caloric irrigation was performed for the right and the left ear at both 44°C and 30°C. Canal function was also evaluated by measuring the vestibular-ocular reflex during a low frequency rotatory test (0.05 Hz, period 20 s). The Video Head Impulse Test (VHIT, Otometrics, see Curthoys & Halmagyi, 1988) [34] was performed with an automatic sensitive video camera system for each of the six canals. Saccular function was tested with Vestibular-Evoked Myogenic Potentials (VEMP). Only patients without significant responses to caloric tests, without response to rotatory test and no VEMP response were included in this study. Moreover, the majority of these patients had also a deficit in high frequency tests (VHIT). Figure 1 shows an example of this test from one patient. The head velocity signal is shown by the blue or orange traces and the eye velocity by the green traces. Eye velocity signals indicate very reduced gain followed by multiple catch up saccades. Participants received general information about the experimental procedures prior to the experiment, but none of them were informed about the specific hypothesis of the study.

Sixteen control adults participated in the present experiment, ages ranged from 22 to 59 years (mean, **31.7±10.5**). No subjects showed visual, neurologic, or psychiatric disorders or received medication.

The eye movement and posture study on patients was carried over a single day, patients came in the laboratory. Because of time limitations and technical problems, the eye movement study was carried on 8 of the patients only (mean age **46.9±12.7 years**), compared with 15 controls investigated in different day (**29.6±7.1, range 22 to 44 years old**). The posturography study was done on the whole groups of patients (11) and controls (16).

The investigation adhered to the tenets of the Declaration of Helsinki and was approved by the local human experimentation committee, the “Comité de Protection des Personnes” (CPP) Ile de France VI (No: 07035), Necker Hospital in Paris, France. Informed written consent was obtained from subject.

### Eye movement study

#### Visual display

**Figure 2:**
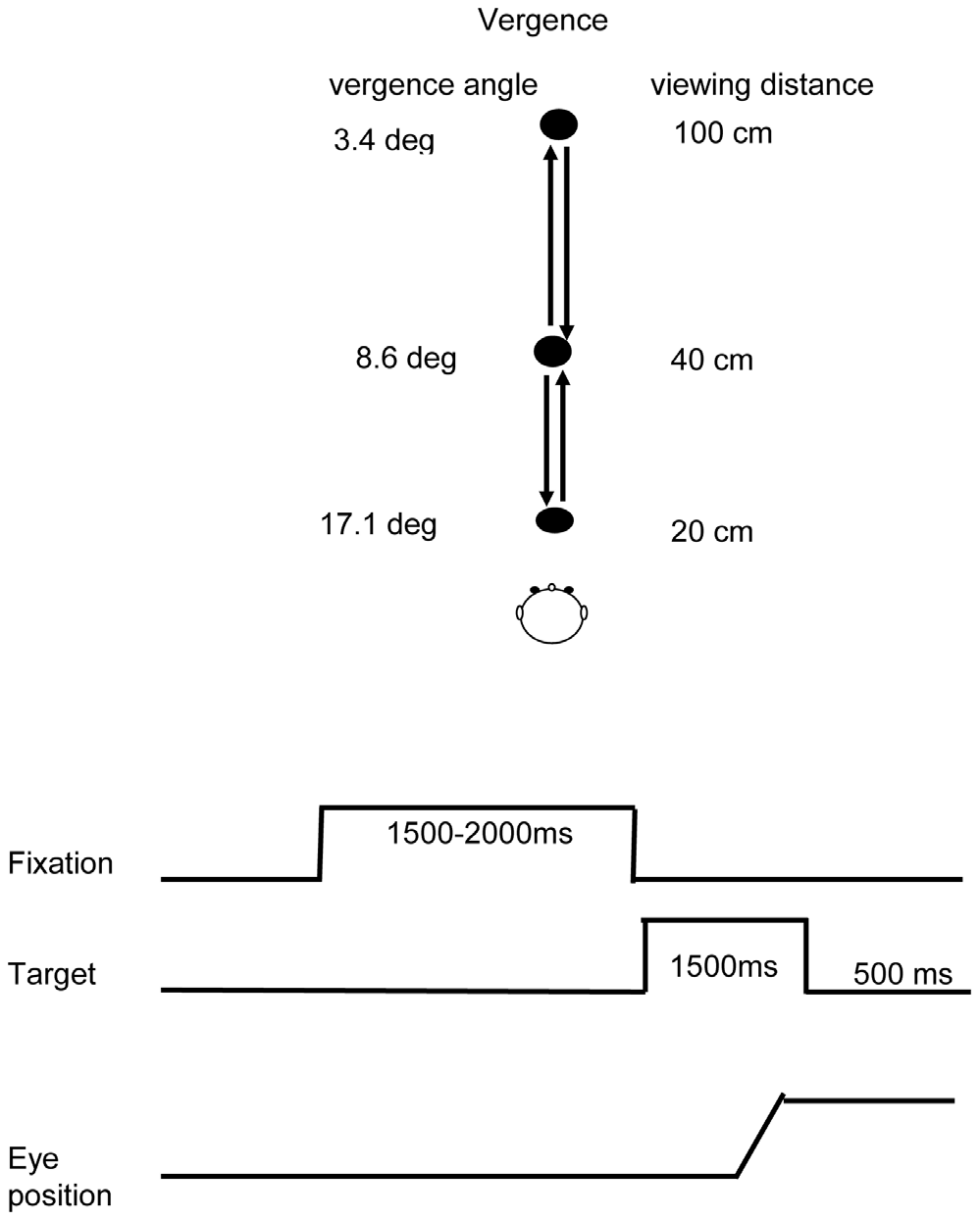
Spatial arrangement for vergence (A): three LEDS aligned along the median plane of the subject: at 20, 40 and 100 cm. The plate of the LEDs was placed at eye level. (B) Paradigm used for the stimulation: the initial fixation LED (always at 40 cm, vergence angle 8.6°) stayed on for a random period of time between 1.5 and 2 s; then the fixation LED was switched off and the target LED was switched on simultaneously. The target LED remained on for 1.5 sec. A black period of 500 ms followed before the next trial. The target LED could be either at 20 cm (requiring an increase of convergence angle from 8.6° to of 17.1°, or at 100 cm, requiring a decrease of convergence angle from 8.6° to 3.4°, i.e. a divergence movement)

The visual display on a horizontal table consisted of circular LEDs (each LED on 2.9 mm, wavelength 636 nm with intensity of luminous 60 mcd) placed at three viewing distance in middle line, 20 cm, 40 cm and 100 cm from the subject. Fixation of these three LEDs requires vergence angle of 17.1°, 8.6° and 3.4°respectively (see Figure 2A). In a dim room, subject was seated in an adapted chair with a chin and frontal rest. He/she viewed binocularly and faced the visual display of the LEDs. Vertically, all target LEDs were placed at eye level.

#### Oculomotor tasks

Each trial started by lighting a fixation LED at 40 cm. The fixation LED stayed on for a random period between 1.5 and 2 sec. The target LED was kept on for 1.5 sec (Figure 2B). Subjects were required to initiate a vergence to the other central target LED as rapidly and accurately as possible. A black period of 500 ms separated trials. Subjects were instructed to use this period for blinks. The total mean length of each trial was about 4 sec. Subject performed one block which contained 20 trials i.e., 10 divergence trials (from 40 cm to 0 cm) and convergence trials (from 40 to 20 cm) interleaved randomly.

A calibration sequence was performed at the beginning of each block; the target made the following predictive sequence for viewing distance at 40 cm: center, 10° to left, 10° to right; the target stayed at each location for 2 sec. From these recordings (four times for each location) we extracted calibration factors.

#### Eye movement recording

Horizontal movements from both eyes were recorded simultaneously with the IRIS, SKALAR device. Eye position signals were low-pass filtered with a cut-off-frequency of 200 Hz and digitized with a 12-bit analogue-to-digital converter and each channel was sampled at 500 Hz.

#### Data analysis

**Figure 3:**
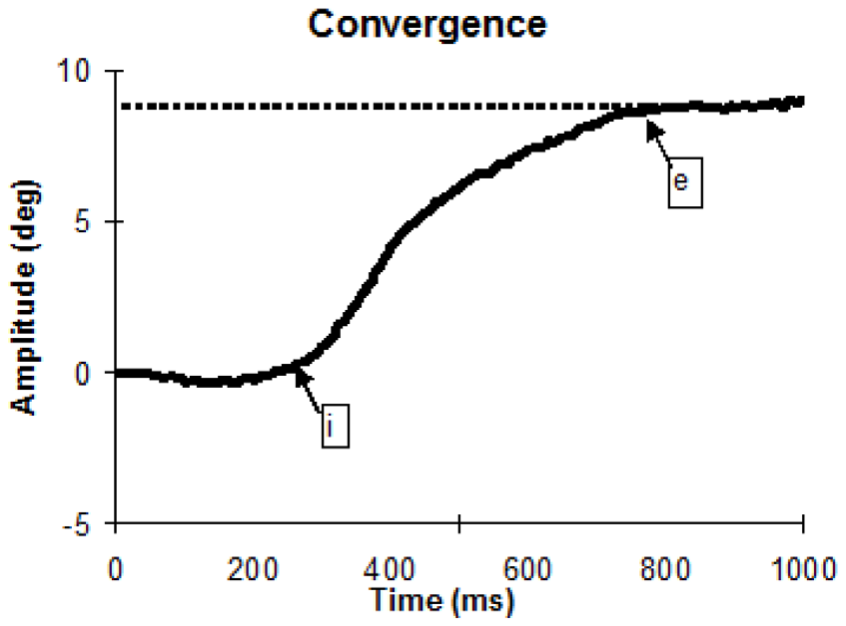
Typical recording of convergence eye movement. Markers ‘*I*’ and ‘*e*’ indicate the onset and the offset of vergence eye movement based on the criterion of 5°/s velocity threshold (see Methods)

From the two individual calibrated eyes position signals we derived the disconjugate signal (left eye - right eye). The eye velocity of either conjugate (saccades) or disconjugate (vergence) signal was computed using a symmetrical two-point differentiator after low-pass filtering with a Gaussian FIR filter with a cut-off frequency of 33 Hz. The initial (‘I’) and ending position (‘e’) of the vergence eye movements were defined as the time point when the vergence velocity exceeds or drops 5°/s (Figure 3). This criterion is standard [35], [36]. The process was performed automatically by the computer, and the verification was made by visual inspection of the individual eye position and velocity trace.

For both convergence and divergence, we measured the latency, i.e. the time between target onset (0 ms) and vergence initiation (marker ‘I’ in Figure 3), accuracy (gain), i.e. ratio of the amplitude of vergence (‘I’ to ‘e’ at position trace, Figure 3) over the amplitude of the target excursion in depth, and the mean velocity, amplitude of vergence and the vergence duration. In addition, amplitude of embedded saccades was also analyzed. Saccades were identified on the basis of velocity and acceleration criteria (eye velocity >35°/s; eye acceleration >1000°/s) [37].

Eye movements in the wrong direction, with latency shorter than 80 ms (anticipation) or longer than 1000 ms or contaminated by blinks were rejected. For control adults five percent of trials and for patients about fifteen percent of trials had to be rejected using these criteria; most individual means were based on 7 measures or more.

#### Statistical analysis

A non-parametric analysis U Mann Whitney Test was performed on individual values of each parameter for convergence or divergence.

### Postural control in quiet stance

#### Procedure

Subjects were required to stand upright and barefoot, with their feet placed side-by-side forming a 30° angle and with their heels separated by 4 cm; they fixated an LED target placed on an horizontal table at eye level in a lighted room. They were asked to maintain quiet stance while fixating the target (i.e. arms held side-by-side, silent with teeth unclenched and normal breathing). Four conditions were run, each lasting 1 min: fixation of an LED at 40 cm requiring sustained convergence of 9°; fixation of an LED at 150 cm requiring reduced convergence (2.3°); this condition was repeated with eyes closed. In the fourth condition, two LEDS, one at 20 and the other at 100 cm, were light on, one after other for 1 sec, causing the eyes to converge then diverge.

#### Posturography

A body fixed sensor (Accelerometer) was used. The accelerometer (Dynaport, MiniMod, McRoberts B.V. The Hague, The Netherlands) was placed at the lower back (L5). The MiniMod makes use of a triaxial scismic acceleration sensor (AXXL202, Analogue Devices, Norwood MA, USA). The sensor's full scale range is ±2 degrees. The frequency response of the sensor is 0–6 Hz (3dB). The sampling frequency is set to 100 Hz.

#### Postural parameters

Parameters analyzed are: the normalized area (in mm^2^/s), the Root-Mean-Square of Medio-Lateral body sway (RMS of M/L in mm), the Root-Mean-Square of Antero-Posterior body sway (RMS of A/P in mm), the RMS of M/L velocity (in mm/s), the RMS of A/P velocity as well as the mean power frequency (Hz). The first three measures describe the stability while the last three concerns mostly the energy used to stabilized the body.

#### Data analysis

We evaluated the Quotient of Romberg (QR) as the ratio of values in the conditions eyes open fixating at 150 cm versus eyes closed; the QR was estimated for each of the parameters listed above. Then we performed a non-parametric analysis U Mann Whitney Test for each postural parameter, comparing the QR of the patients and the controls group.

To evaluate the effects of distance (far, near) and of vergence, we run a two way mixed ANOVA design with the viewing condition (Near 40 cm, Far 150 cm and Vergence movement) as the main factor, and the group (patients and controls) as the inter-subject factor.

## Results

### Eye movement results

**Figure 4:**
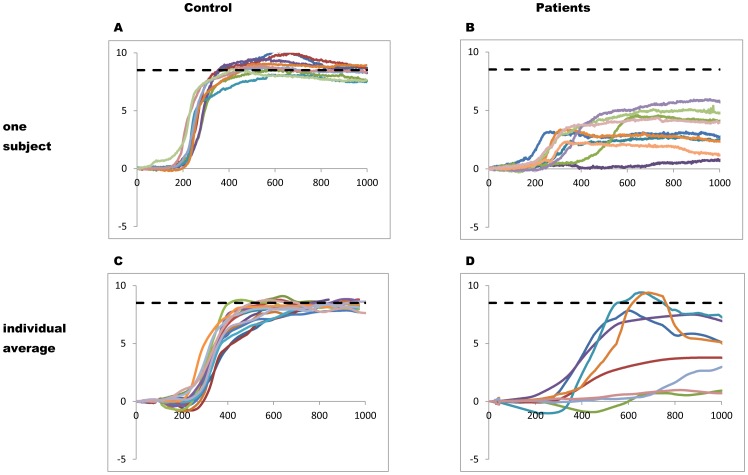
Examples of convergence traces from a healthy subject (A) and from a patient (B) and of convergence traces for all the controls (C) and all the patients studied (D): traces are surimposed in time, target onset was at time zero; the horizontal line indicates the convergence required by the target. Movements from the patients are variable, highly hypometric, slow and not smooth.

Inspection of eye movement traces indicated abnormalities of convergence in patients. Figure 4A and B shows typical examples from a healthy subject and from a patient: convergence traces are highly hypometric, slow and less smooth in the patient. Figure 4C shows average traces from each of the controls and Figure 4D shows average traces for each of the patients. The majority of patients show highly disturbed convergence trajectories. The three patients presenting higher amplitudes still show abnormal trajectories relative to those of controls. Next, we will report quantitative results.

#### 1.1 Latency

**Figure 5:**
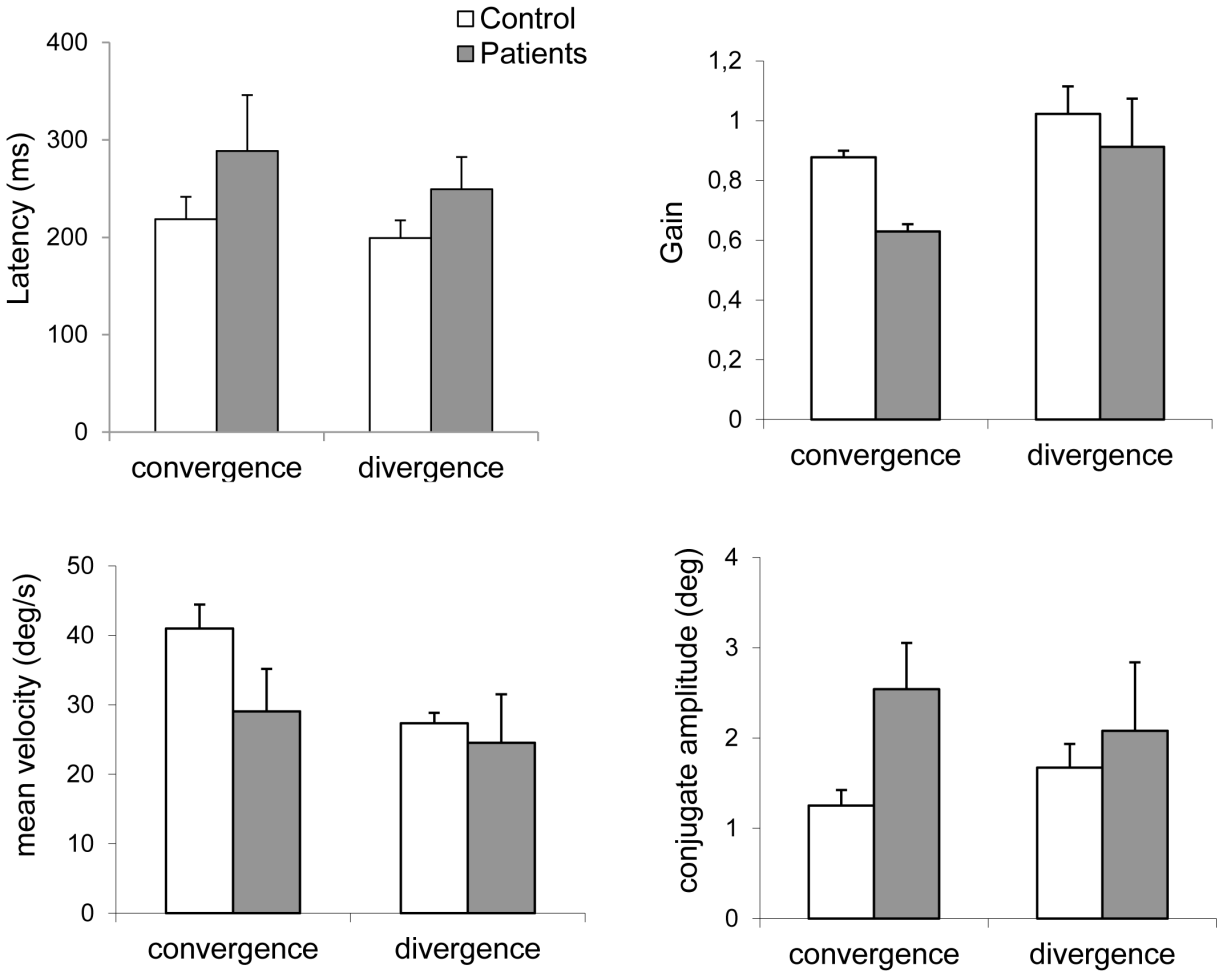
Group mean values for different parameters of convergence and divergence movements in controls and in patients: (A) latency, (B) gain i.e. movement amplitude/target requirement, (C) mean velocity, i.e. amplitude/duration, (D) amplitude of saccade intrusions during vergence movements, i.e., conjugate components. Vertical bars indicate standard errors of the means. Asterisks indicate statistically significant differences between patients and controls that occur specifically for convergence

The group mean latencies and the standard deviation are shown for convergence and divergence in controls and patients in Figure 5A. The U Mann Whitney test applied on the latency values shows no effect of group (U = 38, p = 0.16 for convergence; U = 31.5, p = 0.14 for divergence).

#### 1.2 Gain

Figure 5B shows the group means gain values of vergence with the standard deviation in control and patients respectively. The U Mann Whitney test applied on the gain values shows significant lower gain for convergence in patients than in controls (U = 19, p<0.05), but no group difference for divergence (U = 38, p = 0.31).

#### 1.3 Mean velocity

Figure 5C shows the individual mean velocity with the standard deviation for convergence and divergence in controls and patients, respectively. The U Mann Whitney test applied on the gain values shows significant lower mean velocity for convergence in patients than in controls (U = 18, p<0.05), but no group difference for divergence (U = 30, p = 0.11).

#### 1.4 Amplitude of saccade intrusion

Figure 5D presents the group mean amplitude of the subgroup of embedded saccades during vergence. The U Mann Whitney test applied on the amplitude of saccade intrusion shows significant higher values for convergence in patients than in controls (U = 21, p<0.05), but no group difference for divergence (U = 40, p = 0.0.38).

### 2. Posture measures

#### 2.1 Quotient of Romberg

**Figure 6:**
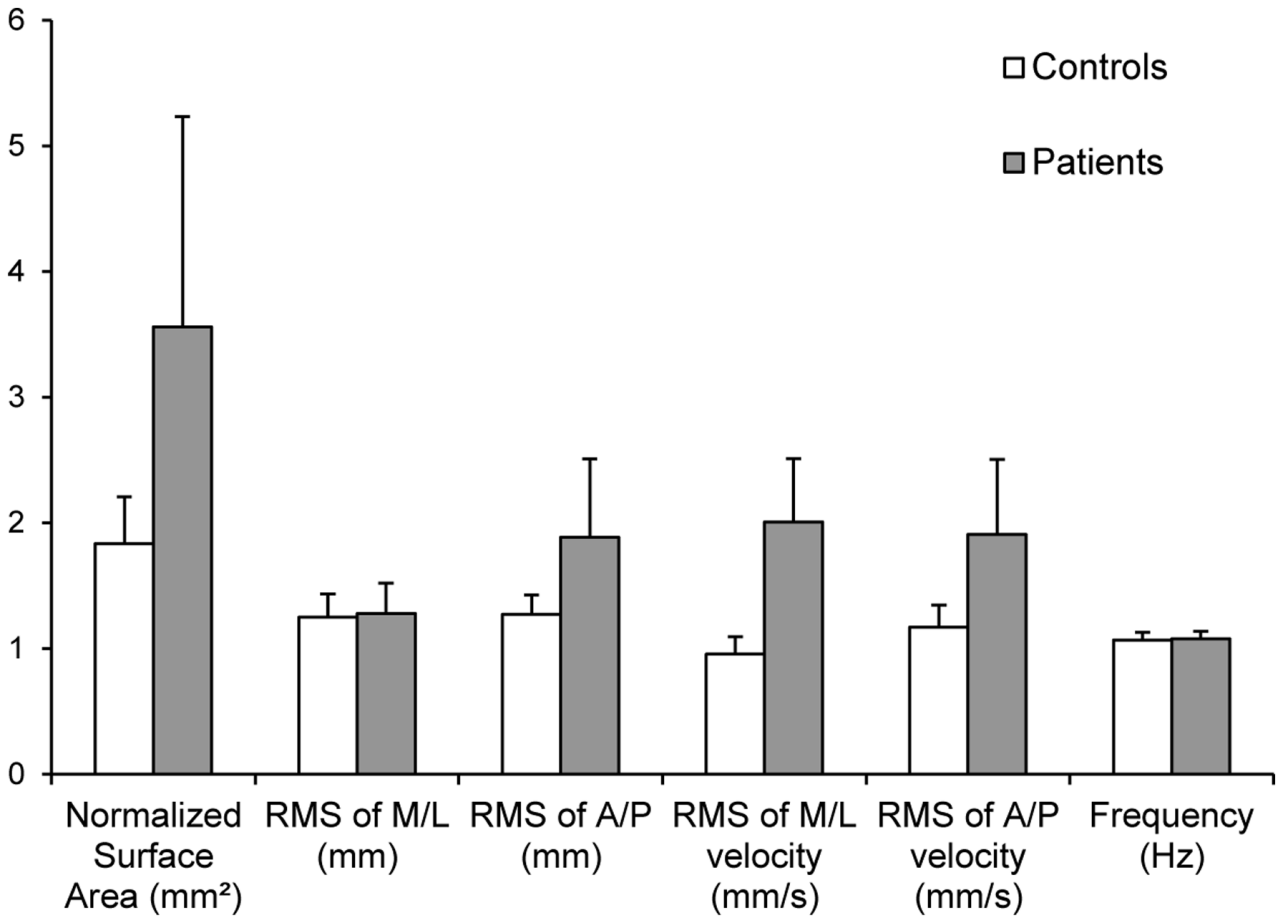
The groups mean Quotient of Romberg, i.e., the ratio of postural value in eyes closed versus eye open condition for each of the postural parameters indicated on the X axis. Vertical bars are standard errors. For the majority of parameters QR are higher in patients than in controls, but also highly variable,; the results suggest that with eye closed postural stability is more perturbed in patients relative to eyes open.

Figure 6 show the Quotient of Romberg (QR) for each parameter for the patient and the control groups. The QR of the patients is higher for the RMS of M/L velocity (mean: 2.01±1.67 for patients and 0.95±0.58 for controls), for the RMS of A/P velocity (mean: 1.91±1.99 for patients and 1.17±0.72 for controls), for the RMS of A/P (mean: 1.88±2.06 for patients and 1.27±0.63 for controls). For the normalized surface area the difference is even higher (mean: 4.33±5.63 for patients and 1.83±1.54 for controls). Yet, due to high variability in patients, there was no statistically significant difference between the two groups; all values of the Mann-Whitney test failed significance. Normalized Surface area (p = 0.4), RMS of M/L (p = 0.21), RMS of A/P (p = −0.35), RMS of M/L velocity (p = 0.11), RMS of A/P velocity (p = 0.65) and Frequency (p = 0.94).

#### 2.2 Far-close-vergence

**Figure 7:**
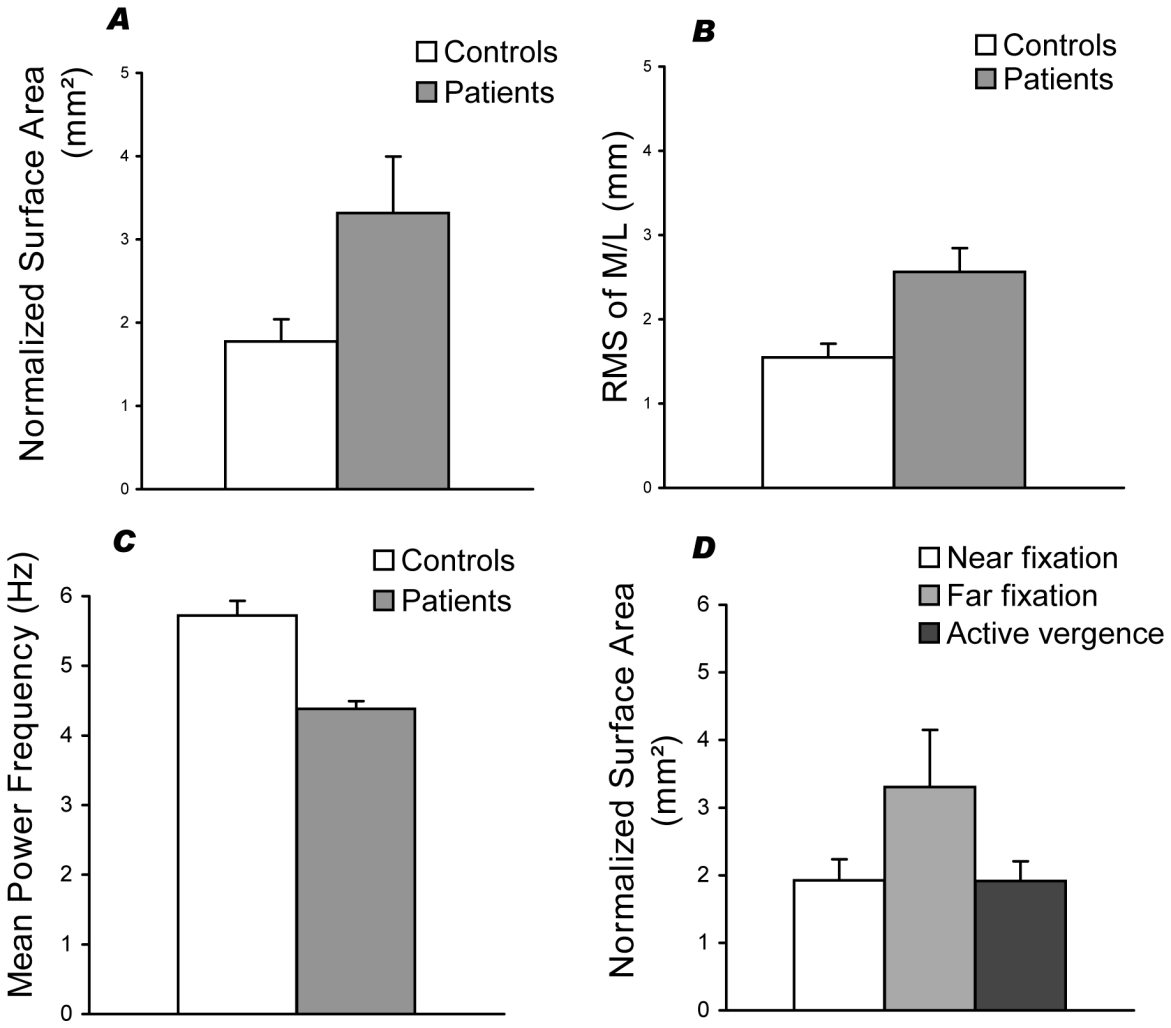
Group mean values of postural parameters together with standard errors in patients and controls: (A) normalized surface area, (B) RMS of medio-lateral body sway, (C) mean power frequency. All these parameters, grouped over all tested conditions are statistically different between controls and patients (see asterisks). (D) Group mean values together with standard errors (vertical bars) for the three conditions, sustained fixation at near, at far, active vergence eye movements. Results from patients and controls are grouped. Fixating at far is associated with significantly higher instability with respect to each of the other two conditions.

***2.2.1 Group effects.*** There was a main effect of the group for the parameter surface (F_(1,26)_ = 4.24, p<0.049). The post-hoc test showed that the surface was higher for the patients than for the controls (p<0.049) (See Figure 7A).

There was also an effect of group for the RMS of M/L (F_(1,26)_ = 4.24, p<0.049), the post-hoc test showing again that the patients had a higher RMS of M/L than the controls subjects (p<0.013, see Figure 7B).

Finally, there was a group effect for the mean power frequency parameter (F_(1,26)_ = 4.24, p<0.049). The post-hoc test showed that the mean power frequency was smaller for the patients than the controls (p<0.0062) (See Figure 7C).

There was no other effect of group on the other postural parameters, and no interaction with conditions.

***2.2.2 Distance - Vergence effects.*** There was a main effect of the viewing condition for the parameter Surface (F_(2,52)_ = 4.31, p<0.019). The post-hoc test showed that the Surface was smaller for all subjects at near distance, compared to a fixation at far distance (p<0.014). In the same way, the surface in the vergence movement condition was smaller for all subjects than in the far fixation condition (p<0.01) (See Figure 7D). There was no other effect of the viewing condition on the other postural parameters.

## Discussion

This study brings important new findings both for vergence and postural control in subjects with idiopathic bilateral vestibular loss. The study was driven by the general idea of a symbiosis between vergence vestibular and postural functions. The eye movement study shows a deficit specifically for the convergence trajectory and its accuracy. The posture study focused on the relation with vergence. It confirmed posture instability relative to controls for all conditions, but showed normal relation to vergence, i.e. better stability with convergence while fixating at near, or while making active vergence movements. These results will be discussed below.

### Abnormality of convergence

Divergence eye movements were normal in our patients. Convergence had normal latency as well. The deficits were highly specific, e.g. high hypometria of convergence, low mean velocity and increase amplitude of saccade intrusions. The accuracy (mean gain value) was as low as 0.6 in patients; such low gain of convergence cannot be explained by possible difference in age between patients and controls. Indeed, Yang et al. [38] using a similar set up for testing vergence showed that the accuracy was around 0.8 even for elderly of mean age 70±11 years. Low accuracy, low velocity and saccade intrusions with large amplitudes observed in the convergence traces in our patients rather reflect dysfunction of the areas controlling convergence including brainstem areas and perhaps the cerebellum. In the theoretical framework of saccade-vestibular function symbiosis, we argue that loss of vestibular input results to weakening of convergence eye movements. Indeed, it is known that in human, vestibular projections reach many brain areas, such as the parietal-insular-vestibular cortex, the somatosensory cortex, the area MST, the intraparietal sulcus and the hippocampus [39]. Many of these areas (e.g. parietal, temporal) are also involved in vergence control [40]. Interactions of vestibular and vergence signals in the brainstem generators and the cerebellum are also known to exist [40].

The question arises why the deficit is specific to convergence. Perhaps this is because of the higher interaction between convergence and vestibular function due to the scaling of the gain of VOR for near distance [19], [25], [40], [41]. The observations of specific convergence deficits is also in line with studies mentioned in the Introduction indicating distinct control in the mesencephalic reticular formation for convergence and divergence [12], or specific cortical EEG activation for convergence and divergence [3].

Another important question is to what extent the deficit in our patients was specific to vergence or could affect other movement such as saccades. We evaluated the properties of saccades during the calibration procedure (targets at 10°, see Methods). All parameters were found to be normal: mean size of saccades was 9.59±1.18, their mean duration was 49±10 ms and their peak velocity was 296±98 deg/sec. All these values were within normal range (see cross-study average normal values, in Leigh and Zee 2006, pp. 111) [40].

### Posture

The high values of the Romberg Quotient for the patients, particularly for the surface parameter, even though not statistically different from that of controls, indicate the increased visual dependency due to loss of vestibular function. This observation is in line with clinical criteria used for evaluating vestibular loss and with extensive literature on posture abnormalities; for a recent review and model see Mergner et al. (2009) [41]. According to these authors, persistent difficulties in posture when eyes are closed are attributed in difficulties in performing decomposition of the force cues into its constituents (e.g. the so called somatosensory graviception).

The novelty of the present study was the examination of postural performances of patients in relation to vergence. Relative to controls, the results show larger instability in patients in terms of surface area and RMS of M/L, regardless of the vergence condition (far, near, active vergence). Could instability relative to controls be related to age (as patients were older than controls)? The answer is no because Kapoula and Lê [33] have shown that for healthy subjects of average age 61 years postural stability was similar to that of younger adults (average age 25 years); the only difference was in terms of variance of speed of body sway which was higher in the older subjects. Variance of speed is believed to reflect the energy needed to maintain postural stability. The patients studied here showed larger surface and larger RMS of medio-lateral body sway indicating instability *per se* that could result from the lack of vestibular input. In terms of energy, the patients also showed significantly decreased mean power frequency, indicating that the lower part of the spectrum frequencies was higher; this could happen if the high frequency signals are removed or if the amplitude of a frequency in the signal becomes lower. The most likely interpretation is the later, e.g. reduction of the amplitude due to the absence of vestibular active controller. This observation provides further information confirming postural abnormality in the frequency domain.

The most important result is the effect of depth and related vergence angle: similarly to controls, patients showed better postural stability while fixating at near than at far. Moreover, active vergence movements also provided better postural stability, similar to that for sustained fixation at near distance. The results suggest that sustained convergence and active vergence eye movements are both beneficial for postural regulation in patients; controls studied here showed similar benefit. The results are in line with those previously reported by Kapoula and Bucci [42] for dyslexic children, and Gaertner et al. [43] in strabismus children. The hypothetical mechanism of action of vergence on posture has been discussed elsewhere [42]. Briefly, convergence can act on posture via multiple mechanisms: attention which is intimately linked to converging of the eyes [3], via synergy between extraocular and neck muscles [44] and via the vestibular system [20]. Given the multiple mechanisms via which vergence can influence posture, it is not surprising that the effect is also seen in patients. Thus, even though not perfectly functioning, the vergence eye movements could still be beneficial for postural control in such patients. The interaction between vergence and vestibular function could be reciprocal e.g. vergence would act on vestibular function (action still possible inpatient) and vestibular function would act on vergence (action lost in patients).

### Clinical implications

This study shows specific abnormalities of convergence in patients with idiopathic bilateral vestibular loss. It also shows that postural stability is in general less good in patients but still when their eyes are converging in sustained fixation or while making actively convergence-divergence eye movements, their postural performance is better. Clinically, although the patients had normal visual acuity and normal visual findings, including normal stereopsis (<60”of arc) some of the patients described difficulties in our active vergence test.

Given that convergence is essential for reading and other activities at near vision, the present study suggests, in line with what some patients reported, particular difficulty for moving or working at near space. Yet, as vergence can improve postural control via multiple mechanisms including the possibility of action on vestibular function we suggest more careful exploration of vergence in such patients; most important, active rehabilitation methods based on body-vergence training stimulated together. Ciuffreda *et al.* [45] observed lasting vergence abnormalities in a variety of patients even those with mild acquired brain injury; on the other hand Ciuffreda *et al.* [46] showed the efficiency of vision therapy including eye movement exercises, as considerable residual neural plasticity exists.
